# Particle-Based Microarrays of Oligonucleotides and Oligopeptides

**DOI:** 10.3390/microarrays3040245

**Published:** 2014-10-28

**Authors:** Alexander Nesterov-Mueller, Frieder Maerkle, Lothar Hahn, Tobias Foertsch, Sebastian Schillo, Valentina Bykovskaya, Martyna Sedlmayr, Laura K. Weber, Barbara Ridder, Miriam Soehindrijo, Bastian Muenster, Jakob Striffler, F. Ralf Bischoff, Frank Breitling, Felix F. Loeffler

**Affiliations:** 1 Karlsruhe Institute of Technology, Hermann-von-Helmholtz-Platz 1, 76344 Eggenstein-Leopoldshafen, Germany; E-Mails: frieder.maerkle@kit.edu (F.M.); lothar.hahn@kit.edu (L.H.); tobias.foertsch@kit.edu (T.F.); sebastian.schillo@kit.edu (S.S.); valentina.bykovskaya@kit.edu (V.B.); martyna.sedlmayr@kit.edu (M.S.); laura.weber@kit.edu (L.K.W.); barbara.ridder@kit.edu (B.R.); miriam.soehindrijo@kit.edu (M.S.); bastian.muenster@kit.edu (B.M.); jakob.striffler@kit.edu (J.S.); frank.breitling@kit.edu (F.B.); 2 German Cancer Research Center, Im Neuenheimer Feld 280, 69120 Heidelberg, Germany; E-Mail: r.bischoff@dkfz.de

**Keywords:** high-throughput, combinatorial synthesis, beads

## Abstract

In this review, we describe different methods of microarray fabrication based on the use of micro-particles/-beads and point out future tendencies in the development of particle-based arrays. First, we consider oligonucleotide bead arrays, where each bead is a carrier of one specific sequence of oligonucleotides. This bead-based array approach, appearing in the late 1990s, enabled high-throughput oligonucleotide analysis and had a large impact on genome research. Furthermore, we consider particle-based peptide array fabrication using combinatorial chemistry. In this approach, particles can directly participate in both the synthesis and the transfer of synthesized combinatorial molecules to a substrate. Subsequently, we describe in more detail the synthesis of peptide arrays with amino acid polymer particles, which imbed the amino acids inside their polymer matrix. By heating these particles, the polymer matrix is transformed into a highly viscous gel, and thereby, imbedded monomers are allowed to participate in the coupling reaction. Finally, we focus on combinatorial laser fusing of particles for the synthesis of high-density peptide arrays. This method combines the advantages of particles and combinatorial lithographic approaches.

## 1. Introduction

The necessity of studying a large variety of molecules in a high-throughput manner, mainly triggered by biological research in the early nineteen nineties, has led to the development of different microarray approaches. The most famous among them are the SPOT synthesis of oligopeptides invented by Ronald Frank [1,2], initially commercialized by the company, JPT (Germany) [3], and the lithographic method for combinatorial synthesis of oligonucleotides [4], commercialized by Affymetrix (USA) [5]. Both methods are based on the solid-phase combinatorial *in situ* synthesis of bio-oligomers with selective deposition of suspended monomers onto synthesis substrates.

The early nineteen nineties were also highlighted with the appearance of an industrial particle-based method to generate great molecular diversity: the combinatorial split-mix synthesis [6,7]. Due to advances in robotics, the Merrifield synthesis [8,9] was routinely performed on beads, made of cross-linked polystyrene, with a diameter of 200–500 µm. This bead-based combinatorial peptide library is synthesized on a large number of beads: in each synthesis cycle, the beads are first split into 20 equal portions. Then, one of the 20 amino acid monomer types is coupled to each portion of beads. Finally, all 20 portions are mixed again. Sequential elongation of the peptide chains is realized by simply repeating these steps of the cycle. The split-mix synthesis is the most efficient and quickest procedure to generate an astronomically large diversity of combinatorially assembled molecules. Thereby, nearly every bead displays a different peptide, but always only one kind of peptide per bead. Having a superior diversity generation rate, the split-mix synthesis requires, however, labor-intensive encoding or decoding to recover the amino acid sequences on those beads showing biological activity. Obviously, this drawback is overcome by the array approach, where the sequence can be easily decoded by the position of the molecules.

Unsurprisingly, the two different approaches—arrays and combinatorial functionalization of particles—have met each other in novel high throughput screening technologies. Indeed, most of the following particle-based array systems profited from the efficiency of generating molecular diversity with particles and from the patterning of particle positions in array format. In this review, we first describe the appearance of oligonucleotide bead arrays in the late 1990s. This impressive example of particle-based arrays enabled high-throughput oligonucleotide analysis and had a large impact on genome research. Furthermore, we discuss the advances in the particle-based approach towards peptide array fabrication. A promising tendency is the use of particles as amino acid carriers for the combinatorial synthesis of peptides. For a detailed description of the modern non-particle methods of peptide array synthesis, we refer to the review of Assaf Friedler [10].

## 2. Particle-Based Oligonucleotide Arrays

The emergence of high-density oligonucleotide arrays was triggered by several important factors: First, oligonucleotides play an outstanding role in life sciences, due to their function of storing complex biological information. The human genome, for instance, comprises more than three billion nucleotides, also called base pairs (bp). The ambitious goal to sequence such a large number of base pairs could only be realized by means of novel high-throughput methods. The high-density array concept with an efficient registration of binding signals in an array format presented itself at that time as the method of choice for oligonucleotides. In addition, due to the progress in nucleotide technology, three important methods became available: the generation of large oligonucleotide libraries, the decoding of oligonucleotides by fluorescent labeling and the synthesis of one compound per bead. In this section, we consider several examples of particle arraying for the assembly of oligonucleotide arrays or for high-throughput oligonucleotide sequencing and discuss the emulsion polymerase chain reaction (ePCR) [11].

### 2.1. Illumina Arrays

The company, Illumina, Inc. (San Diego, CA, USA), has attained a leading position in the field of oligonucleotide analysis [12,13]. Since its initial public offering in July, 2000, the company experienced rapid growth and became one of the leading manufacturers of oligonucleotide arrays and sequencers. Their unique particle-based technology was one of the important factors that lead to this unprecedented success. Illumina, starting with single nucleotide polymorphism genotyping, currently offers microarray-based products and services for an expanding range of genetic analysis sequencing, gene expression and protein analysis.

**Figure 1:**
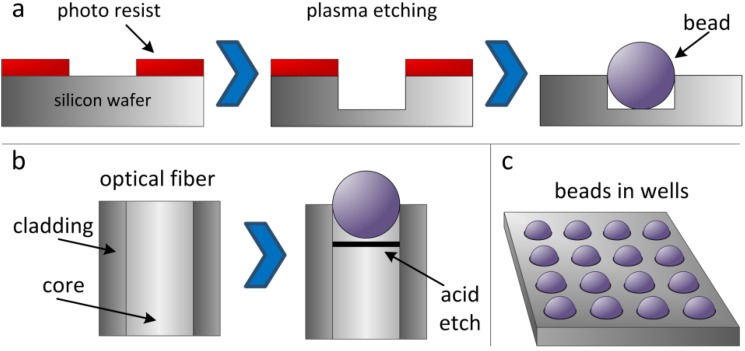
Assembly of particle-based arrays in microstructured cavities on a silicon wafer (**a**) or an optical fiber (**b**), yielding the microbead array (**c**).

The particle-based approach was invented by David Walt at Tufts University (USA) and has been used as a platform for a wide range of assays [14,15]. The original idea was to use the particles as biosensors [16,17]. Particles were deposited on the microstructured cross-section of a waveguide and could be illuminated for further analysis. Very soon, this technique found its application in the assembly of oligonucleotide arrays: First, a library of oligonucleotides was synthesized using standard technologies on beads. Each oligonucleotide is synthesized in a larger batch, so that one molecule type is coupled to several beads. This step is crucial for a simple and high-quality readout, because each bead represents a unit of one type of combinatorial molecule, whereas each bead features hundreds of thousands of copies of this combinatorial molecule. Illumina uses three-micron silica beads (or other materials, e.g., polystyrene), which are randomly scattered across micro-etched substrates (e.g., optical fibers or silicon wafers). These microstructured surfaces feature an array of microwells with a uniform spacing of approximately 5 µm. The geometry of a well is set up to catch a single particle (Figure 1a,b). The beads in the microwells are kept in place by strong adhesion forces, so that the particle arrays can be easily handled in microfluidic systems without causing beads to detach from the microwells.

Now, different beads with different predefined oligonucleotide sequences are randomly assembled in the microstructures. In the case of arrays on microstructured silicon wafers (Figure 1a), decoding of the molecule type (*i.e.*, bead type) is conducted by using a series of decoding hybridization steps with conventional fluorescence techniques [18,19]. For easier fluorescence readout, Illumina also used multicore optical imaging fibers (Figure 1b), which are etched such that about 50,000 beads fit into the resulting microwells on the tip of the multicore fiber. The light propagating through the fibers is collected to decode and analyze the beads.

**Figure 2:**
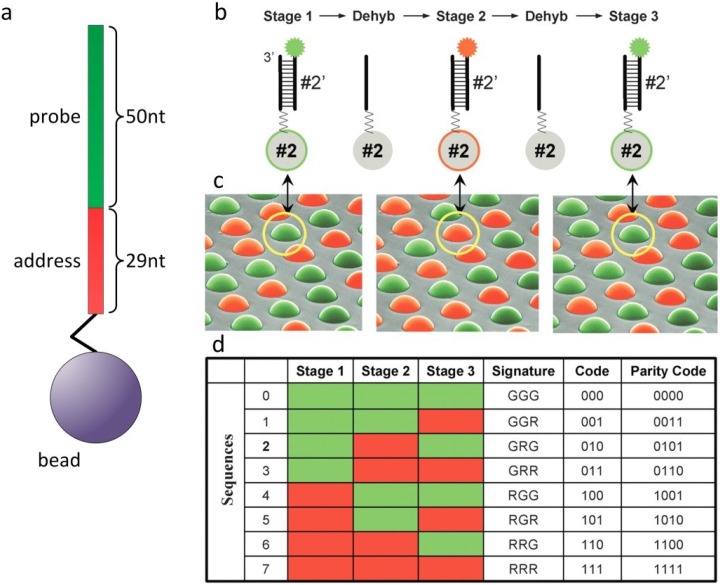
Decoding of different sequences on a randomly ordered bead array. (**a**) A 79bp oligonucleotide with the 29bp address segment and the 50bp probe segment, linked to a bead. (**b**) In the first step of decoding, a set of complementary decoding nucleotides is labeled green and red. Upon hybridization, Sequences 0–3 fluoresce green, while Sequences 4–7 fluoresce red. After dehybridization (Dehyb), another decoding solution is hybridized with a different set of decoding nucleotides labeled in green and red. Three iterative hybridization-dehybridization steps render unique codes for every bead in this example nucleotide sequences GGG, GGR, GRG, GRR, RGG, RGR, RRG, RRR. Reprinted with permission from Cold Spring Harbor Laboratory Press: Genome Research [4].

During synthesis of the oligonucleotides, part of each sequence is reserved for decoding (Figure 2a). For gene expression analysis, Illumina uses a 79-bp oligonucleotide, with the 3' located 29-bp segment of the oligonucleotide as the address and the 50-bp segment at the 5' end for sample analysis. Differential labeling uses three states: carboxyfluorescein (FAM) labeled (green), cyanine 3 (Cy3) labeled (red) and unlabeled (black). While decoding, a bead can be in three different states: green, red or black. The advantage of a stepwise decoding is that only a small number of fluorophores and sequential decoding steps allow for an exponential number of codes: three decoding steps and three labeling states already yield 27 different codes. The process starts by hybridizing labeled decoding nucleotides with the address segments on the beads at high concentrations, which allows for rapid hybridizations, followed by washing to remove non‑specific signals and the background (Figure 2b,c). After the fluorescence readout, several rehybridization steps with other decoding nucleotide sets are performed, until there is sufficient data to unambiguously determine the identity of each bead (Figure 2d). The accuracy of decoding is estimated to be 99.99%. Because of the statistical fabrication process, each bead type occurs on average about 30 times on one array. Therefore, the impact on assay results with an error rate of one in 10,000 decoded beads is negligible.

### 2.2. Particle-Based Emulsion PCR

**Figure 3:**
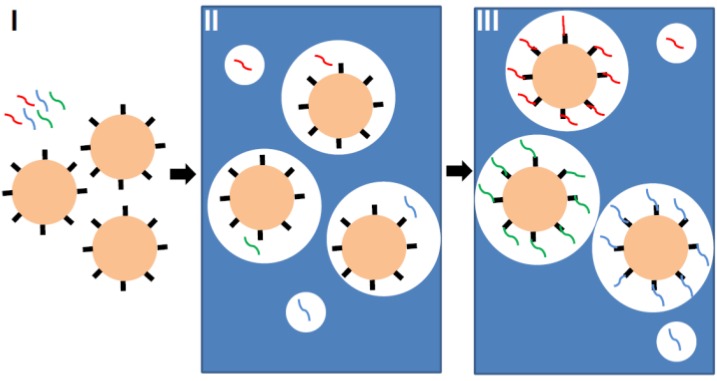
Principle of emulsion PCR. First, beads with coupled primers and template DNA (red, blue and green lines) are prepared (Step I). Then, an aqueous solution containing all of the necessary components for PCR is mixed with oil and detergent to create microemulsions (Step II). The dilution is thoroughly adjusted, so that each aqueous compartment (white circle in blue oil phase) contains at maximum one bead and one template. Finally, the microemulsions are temperature-cycled as in a conventional PCR (Step III), and the bead-bound oligonucleotides are synthesized (also see [11]).

Emulsion polymerase chain reaction (ePCR) is one of the key elements that enabled the production of particle-based high-density oligonucleotide arrays. The goal of ePCR is to produce beads, each of them carrying only one type of oligonucleotide compound (Figure 3). First, beads with coupled primers, template DNA and an aqueous solution, containing all of the necessary components for PCR, are mixed with oil and detergent to create microemulsions. The dilution is thoroughly adjusted, so that each aqueous compartment contains at maximum one bead and one template. The necessary condition for the “one bead one molecule” regime is a low template concentration and the preparation of more uniformly-sized aqueous compartments, which can be generated by applying sonication or pressure-driven emulsifiers. Finally, the microemulsions are temperature-cycled as in a conventional PCR, and the bead-bound oligonucleotides are synthesized. After PCR, templates are denatured and removed, and a bead enrichment step is performed by centrifugation to separate beads with successful oligonucleotide synthesis from non-templated beads [18]. Each bead can hold up to 100,000 amplified copies of one specific sequence. Emulsion PCR is considered as the method with minimal loss in template molecules. After successful amplification and enrichment of beads, millions of them can be assembled in array format for further analysis. Different approaches were reported: beads were assembled in microfluidic channels [20], immobilized in a polyacrylamide gel on a standard microscope slide [21], chemically cross-linked to an amino-coated glass surface [22] or deposited into individual wells [23].

New particle-based PCR methods for high-throughput screening have been reported, e.g., a low-volume PCR amplification, immobilizing the PCR product on arrayed DNA capture beads in microwells [23]. In this case, the particle-based PCR is performed similarly to PCR in solution, except that DNA capture beads are captured in wells prior to amplification. The amplification is conducted with the PCR reaction mix containing no template.

### 2.3. Particle-Based Arrays for Sequencing

In this paragraph, we focus on the methods of particle assembly in arrays, omitting details in sequencing chemistry, which can be found elsewhere [24].

**Figure 4:**
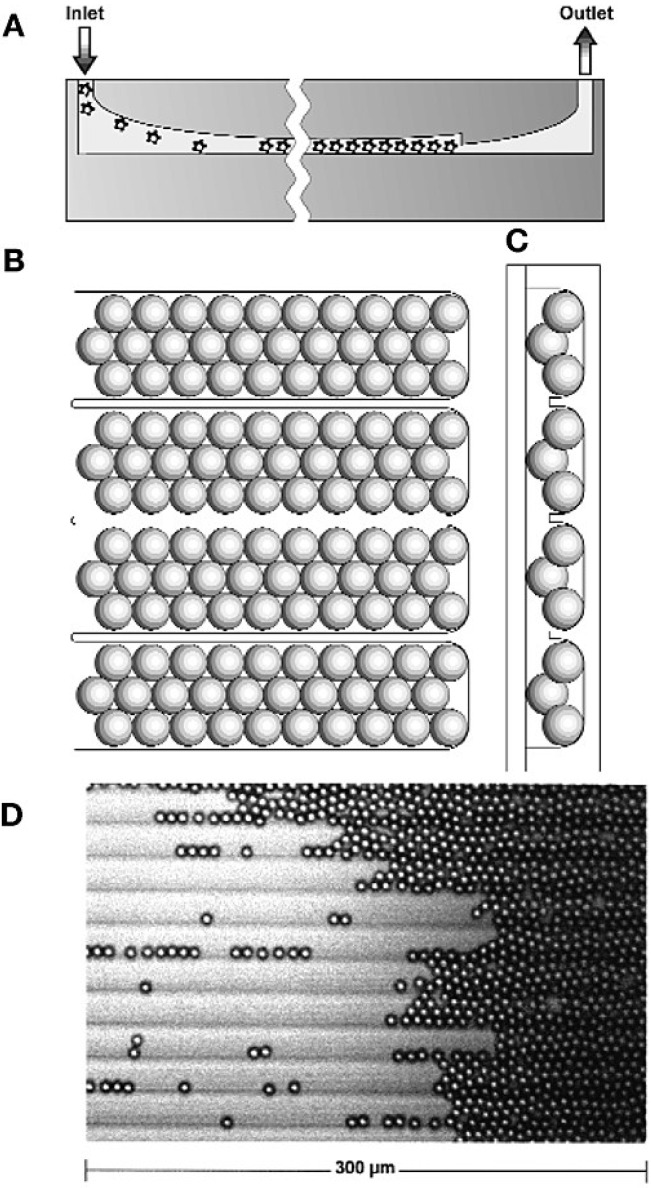
MPSS flow cell design and use. The flow cell (**A**) in longitudinal cross section; (**B**) top view; (**C**) lateral cross-section. (**D**) Assembly of microbeads in grooves (fluid flow from left to right). Reprinted with permission from Macmillan Publishers Ltd.: Nature Biotechnology [20].

One of the first applications of particle-based arrays for sequencing was described by Sydney Brenner and coworkers, who commercialized their massively parallel signature sequencing (MPSS) as the Lynx Megaclone technology [20]. MPSS was developed to ligate many different cDNA fragments onto microbeads, which are then arrayed in a flow cell for sequencing and quantification. The flow cell was fabricated by micromachining a glass plate to form a grooved chamber for immobilizing microbeads in a planar array (Figure 4A–C). Microbeads in solution are loaded into the flow cell through the inlet, travel along the grooves and, finally, pack against a vertical constriction, adjacent to the outlet, to form a quasi-random array (Figure 4D). The sequence signatures are deciphered by the parallel identification of four bases by hybridization to fluorescently-labeled encoders. The authors were able to work with a large amount, up to a million particles each containing 100,000 cloned copies of cDNA from each mRNA molecule of a particle. The raw output of MPSS was reported to be 17–20-bp signature sequences per bead.

In another approach [18], it was found that a simple acrylamide-based gel system developed for compact DNA polymerization in small colonies called “polonies” was easily applied to ePCR beads, resulting in a 1.5-cm^2^ array of disordered, monolayered, immobilized beads. For this purpose, beads are poured in a 5% acrylamide gel onto a silane-treated 40-mm round glass coverslip. The gel geometry is formed using a Teflon-coated glass microscope slide as a template. A slide with a round 14-mm well is thus used to create a circular gel of approximately 30 µm in thickness. Polymerization is slowed down by using reduced amounts of catalyst, so that the beads settle into a single focal plane at the surface of the gel (the coverslip is inverted, so that the exposed gel surface is facing down). A special flow cell was designed to permit sequential biochemistry cycles to be performed upon the beads bound to the bottom of the cover slip. Reagents enter the flow cell one at a time, via the entry port at the center rear of the flow cell. The reagents are routed upwards to the entry bifurcations, which divide the reagents equally among eight parallel lanes. Biochemistry takes place between the reagents and DNA on the surface of beads, which are bound to the glass above each lane. The authors of [22,23] pointed out several disadvantages of the acrylamide-based particle assembling: susceptibility of acrylamide to alkali or dehydration excludes the use of certain reagents (e.g., alcohols, alkaline denaturants and others) during sequencing cycles. In addition, beads within the matrix are not uniformly located in a single focal plane, resulting in diminished performance of microscopy-based data acquisition with lower yield. To overcome these disadvantages, they developed an approach to cross-link amino groups on oligonucleotide-coated beads to amino-silylated glass cover-slips. Then, oligonucleotides (both loaded forward primers and amplified templates) on polony beads are capped with primary amines. Reactive amines on oligonucleotide-coated beads and on glass coverslips are bridged with bivalent amino-ester cross-linkers.

The microstructured surfaces for particle assembling turned out to be useful for sequencing applications. Like in Illumina arrays, self-organization of particles leads to their almost perfect distribution in the wells of the substrate. The microparticles can be assembled in wells either by swiping them across the well [25] or using centrifugation [23].

**Figure 5:**
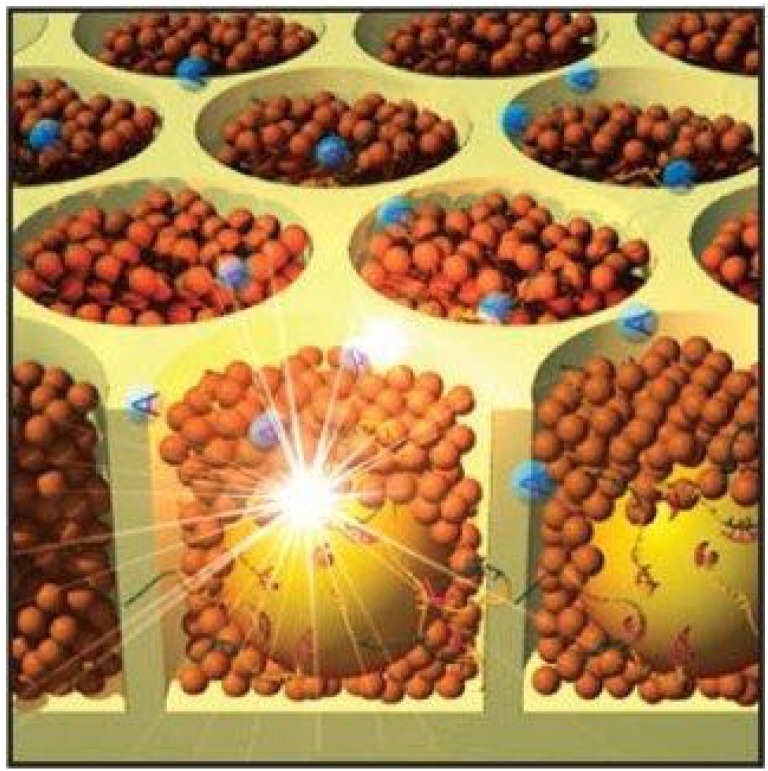
Pyrosequencing in picotiter plate. The beads (yellow spheres) in the wells come into contact with polymerases (brown spheres) and the nucleotide, adenine (blue spheres). The synthesis reaction generates a light signal, which can be detected. By courtesy of Roche Diagnostics Deutschland GmbH.

The company, 454 Life Sciences [26], now part of Roche Applied Science, took advantage of the particle arraying in microwells by developing the 454 sequencing technology, which became commercially available in 2004 (Figure 5). First, the DNA is digested and cleaved into small fragments of about 500 bp. Then, adaptors are ligated to the end of each fragment. In a subsequent ePCR step, these fragments are first attached to the surface of beads using complementary primers and, then, PCR amplified. As a result, the beads will carry many copies of one DNA molecule. Afterwards, the beads are assembled in microwells in an array format of a microstructured surface, containing millions of wells. In the next step, polymerases and nucleotides are added: the nucleotides are derived from pyrosequencing technology [27], generating a burst of light when they are attached by the polymerase. Because only one nucleotide type at a time is present in the system, a camera system can record the light signals, which allows for sequential deciphering of the nucleotide sequence for every bead simultaneously. The approximate maximum of bases that can be sequenced with the 454 technology is 400–600 million bp with a read length of 400–500 bp.

Meanwhile, new particle-free sequencing methods, e.g., “sequencing by synthesis” (Illumina/Solexa, San Diego, CA, USA) [28] and “real-time sequencing” (Pacific Biosciences, Menlo Park, CA, USA) [29], have appeared (also see [24]). These methods feature a significant reduction of sequencing costs, a highly increased throughput capacity (Table 1) and show a large potential for further improvement of these parameters.

**Table 1:** Comparison of high-throughput sequencing technologies (fragment [30]).

Method	Throughput Mb/day	Read Length bp	Quality %	Costs $/Mb
454/Roche	**750**	**~400**	**99.9**	**~20**
Illumina/Solexa	**5,000**	**~100**	**98**	**~0.5**

New particle-free sequencing methods profit from the recent progress in biochemistry, laser and information technologies. In the case of Illumina/Solexa sequencing, a DNA cluster of the same compound is generated on the glass substrate with bridge PCR, called cyclic reversible termination. This technique does not require particles and ePCR and produces oligonucleotide spots of submicrometer size just on the flat glass substrate [31]. The bridge PCR was originally commercialized by Solexa, a spin-off company of Cambridge University. However, the company experienced technical problems in processing the huge amount of data. Illumina acquired the Solexa technology in 2007 and advanced it with its processing competence in particle-based arrays. Up to 2012, this technology has taken 56% of the next generation sequencing (NGS) market, due to its efficiency (the next generation sequencing market expected to grow to $2.7 billion by 2017 [32]). Thus, Roche announced in 2013 that they would abandon the 454 pyrosequencing technology until 2016. As for particle-based 454 sequencing, among the NGS techniques, it still features one of the highest sequencing qualities of 99.9% at a relatively long read length [30].

## 3. Particle-Based Peptide Arrays

The unique feature of oligonucleotides to bind complementary strands allows for their sequencing even at very low concentrations. For instance, in real-time sequencing, it is possible to sequence single oligonucleotides in array format without using pre-amplification [29,33]. In the case of particle-based oligonucleotide arrays, particles are mainly used as carriers of already synthesized combinatorial molecules, so that the sequences on the array are determined just after assembling the particle array. This is yet impossible for peptides. Although efficient mass spectrometric high-throughput methods have emerged in the last decade (e.g., RapidFire technology, Agilent, Santa Clara, CA, USA), in comparison to oligonucleotide sequencing, they are still slow and require a significant amount of prior knowledge about the molecules. Another drawback of mass spectrometry is the problem of distinguishing different sequences of similar amino acids (*i.e.*, the same mass). Without the possibility of rapid *in situ* “sequencing” of small amounts of synthesized peptides on beads in a high-throughput manner, the only way to determine the sequence is to control the particle behavior during combinatorial synthesis of the peptide. This can be realized by labeling particles with tags or barcodes [34]. For instance, suspension microarrays present an embodiment of microarray technology in which the typical spotted planar array is replaced with microspheres with distinct optical properties that can move freely in a solution [35,36].

The development of encoding methods for high-throughput analysis is closely connected to carrier encoding. A prime example is the graphical barcode method relying on the patterning of optical elements on microparticles. This method is based on continuous-flow lithography, which combines particle synthesis, encoding and probe incorporation into a single process to generate multifunctional particles bearing over a million unique codes [37]. However, today’s barcode methods still do not reach the high-throughput capacities known from oligonucleotide screening.

An alternative way, on which we focus in the next section, is the use of particles as monomer carriers for the combinatorial synthesis of peptide arrays. In this case, the decoding of the sequences synthesized is done by selective particle deposition [38].

### 3.1. Amino Acid Particles and Xerographic Methods

**Figure 6:**
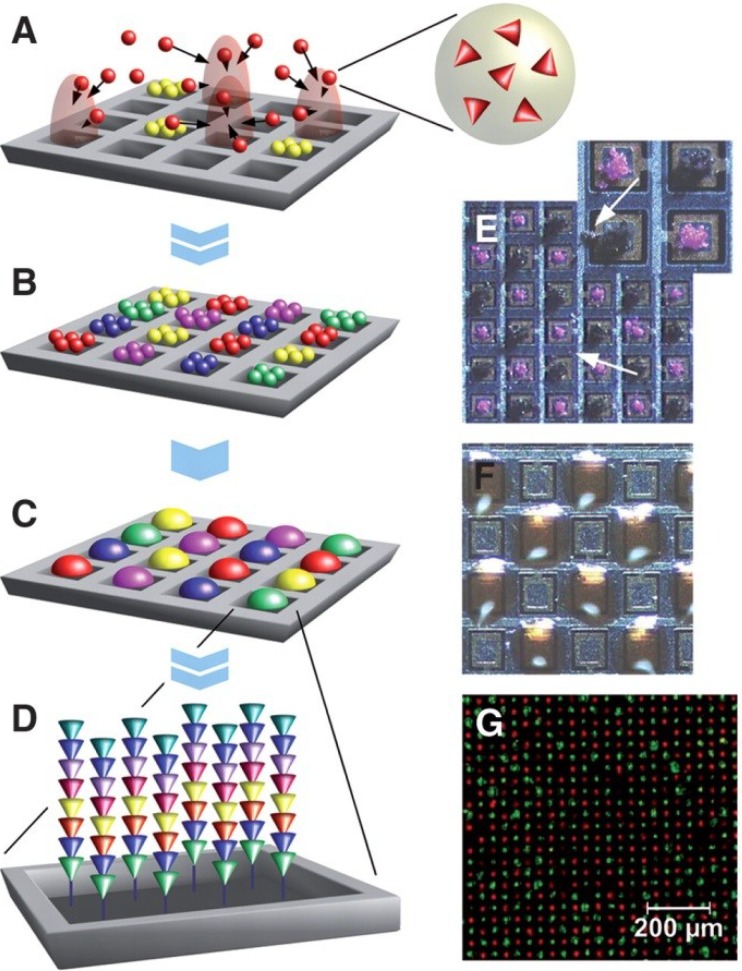
Particle-based synthesis of peptide arrays. Activated amino acids are embedded within particles that are addressed on a chip by electrical fields generated by individual pixel electrodes (**A**). A whole layer of consecutively addressed amino acid particles (**B**) is melted at once to induce coupling (**C**). Repetitive cycles generate a peptide array (**D**). Consecutively deposited, unmelted particles stick to the surface because of strong adhesion forces. Arrows point to wrongly deposited particles (**E**). Melted particles delimit individual coupling areas. For better visualization, pixel areas are overloaded (**F**). Particle-based *in situ* synthesis of chessboard-arranged of amino acid sequences Asp-Tyr-Lys-Asp-Asp-Asp-Asp-Lys (FLAG-tag, green) and Tyr-Pro-Tyr-Asp-Val-Pro-Asp-Tyr-Ala (HA-tag, red) with a density of 40,000 per cm^2^ (**G**). Reprinted with permission from American Association for the Advancement of Science AAAS [38].

Major progress in the xerographic printing techniques (see [39]) in the 1990s has stimulated the idea to exploit the technology for combinatorial synthesis of peptide arrays. In xerography, electrically charged particles are deposited according to locally-generated electrical fields to create images and text. In the 1990s, xerographic devices, such as color laser printers, became an indispensable instrument in offices, due to their reliability and cost-efficiency. In laser printers, the light from an LED line array is used to generate electrical field patterns on an organic-photoconductor drum. This pattern is brought into contact with charged particles to convert the latent electrical image into a corresponding particle pattern on the drum [40]. The first proof of principle peptide array synthesis with charged amino acid particles was shown with a semiconductor chip (Figure 6) [38,41].

A voltage of 100 V was applied to each pixel of the chip, so that it was used to generate patterns of amino acid microparticles with a spot to spot distance of 50 µm. Although only two peptides were synthesized in this experiment, the principle function of the amino acids particles was successfully demonstrated. The composition of the amino acid particles included commercially available activated amino acid monomers (Fmoc transient protection group [42]; OPfP ester activation of the *C*-terminus), a resin (Product number SLEC PLT 7547, SEKISUI CHEMICAL CO., LTD, Osaka, Japan), which served as the matrix for the physical particle stability, charge transfer agents and anti-agglomeration components [38]. The charge transfer agents provide for the stabilization of the electrical charge on the surface of the particles. The amino acid particles, with a relatively narrow size distribution of about 10 µm, are electrically activated to a relatively large charge-to-mass ration of approximately 10^−3^ C/kg by contacting the walls of an acrylic glass cone chamber. After the deposition of the particles, they are melted at temperatures of up to 90 °C. This melted gel-like state allows the monomers to diffuse inside the melted matrix and couple to the solid support according to the classical Merrifield peptide chemistry. Thus, biofunctional xerography exploits the possibility to form peptide bonds in the melted polymer phase at relatively high temperatures.

**Figure 7:**
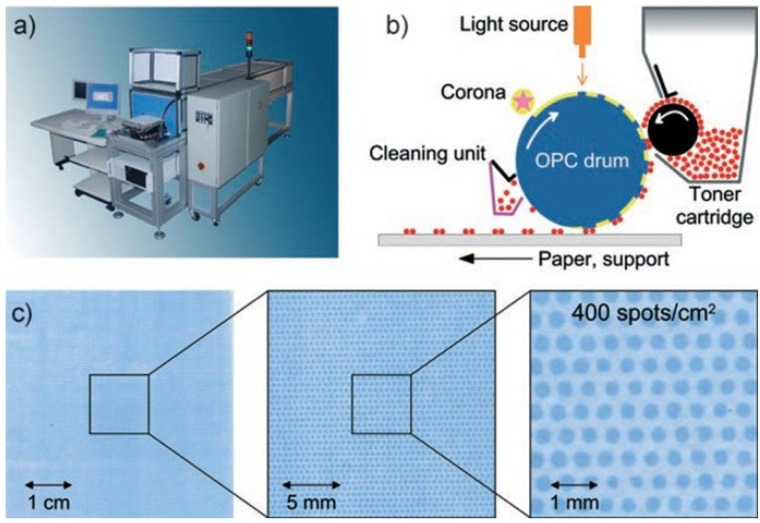
Xerographic laser printing. (**a**) The peptide laser printer with 20 different printing units is shown; the mounting for the support is visible at the front of the printer. (**b**) A corona charges the surface of the OPC drum. A light source (orange) illuminates and neutralizes selected areas. Triboelectrically-charged toner particles are deposited on these neutralized areas, which are then transferred to a solid support. (**c**) An amino acid toner pattern was printed with the peptide laser printer onto a derivatized glass slide and coupled by heating. The free amino groups were stained with 0.1% bromophenol blue. Reprinted with permission from John Wiley and Sons: Angewandte Chemie [43].

The particles developed in [38] were also used in the peptide laser printer [43], which is conceptually based on the color laser printer, OKI C7400, but accommodates 20 instead of four printing units (each of which contains a particular amino acid toner), as well as a drive and a mounting that enable the repeated exact positioning of the solid support (Figure 7a). The laser printing technology has a limited printing resolution of 160,000 spots on an area of 20 × 20 cm^2^ (Figure 7b,c). However, the underlying concept should principally allow for the generation of many millions of different peptides. The laser printing technique was meanwhile commercialized and further developed by PEPperPRINT GmbH, Germany [44].

In a different approach, a CMOS-chip-based xerographic printing machine has been developed to allow for the combinatorial particle deposition on dielectric surfaces with higher spot densities of 10,000 spots/cm^2^ [45]. The high voltage CMOS chip is used as a printing head in this machine. The chip is mounted onto a tilt stage, which can be moved in the x, y and z directions. Combinatorial deposition of particles is conducted in three steps: First, the electrical field pattern is generated on the surface of the chip. Then, the latent image is developed by contacting the chip with the bioparticle aerosol. These two steps can be repeated until the complete chip surface is covered with the desired particle pattern consisting of all 20 different amino acid particles. Finally, the particle pattern is transferred to a dielectric surface, e.g., a glass slide, by applying a homogenous electric field between the chip and an electrode on the backside of the dielectric support. The CMOS-chip-based xerographic printing machine uses 3 µm-sized amino acid particles, which are a factor of three smaller than the particles in the laser printer. This decreasing of the particle size is indispensable for the contamination-free particle pattern generation by exposing the CMOS chip to an aerosol [46,47]. The xerographically-produced peptide arrays have been reported for studies of immunosignaturing effects [48,49], autoimmune antibodies [50,51] and peptide-protein interactions [52].

### 3.2. Combinatorial Laser Fusing of Amino Acid Particles

**Figure 8:**
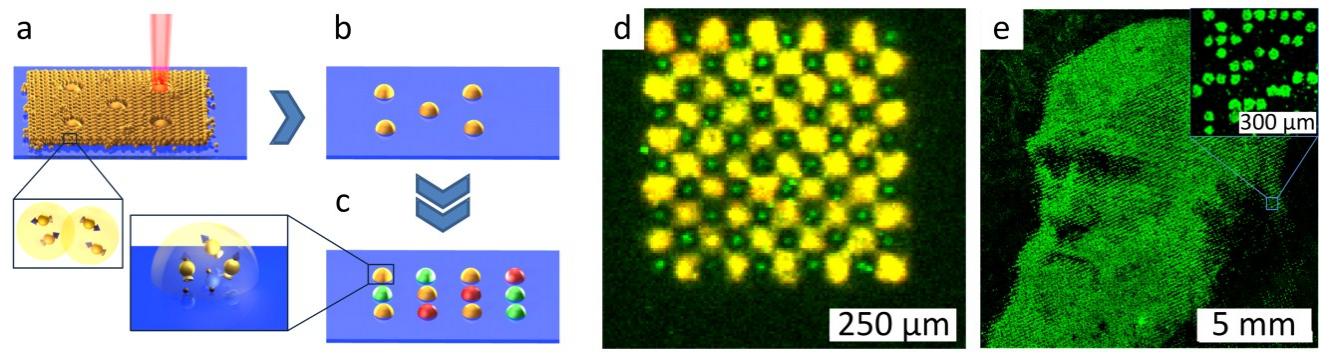
Laser fusing of amino acid patterns. (**a**) A laser beam fuses microparticles with embedded amino acids to an amino functionalized substrate; (**b**) non-fused particles are removed; (**c**) repetitive laser fusing with different particles results in a combinatorial amino acid pattern; (**d**) a fluorescently-labeled peptide array with a spot density of 40,000 spots per cm^2^ produced by combinatorial laser fusing. The pattern of HA and Flag peptides was detected with specific antibodies. The HA peptide was labelled green, and the Flag peptide was labelled yellow. The feature size of HA spots is as small as 10 µm. (**e**) Biotin spots representing the portrait of Charles Darwin detected with fluorescently-labelled streptavidin. The image area is 1.5 × 1.5 cm^2^, which corresponds to a pattern of 90,000 spots (40,000 spots per cm^2^). Reprinted with permission from John Wiley and Sons: Advanced Materials [54].

The emergence of the combinatorial laser fusing method is based on the idea of selectively increasing the adhesion force for combinatorial particle patterning [53]. This modification can be achieved by laser irradiation, which melts and fuses the particles of a homogenous particle layer [54]. The melted particles experience larger adhesion forces than the non-melted ones. The main goal of combinatorial laser fusing was to achieve high spot densities together with time and cost-efficient one-cycle-per-layer coupling of xerographic methods. Figure 8 shows the principle of combinatorial patterning with laser radiation. Repetition of the particle patterning on an amino-terminated substrate, as shown in Figure 8a,b, results in combinatorial patterning of the particle matrix containing different amino acids (differently colored spots in Figure 8c). Heating the substrate for 1 h under inert gas atmosphere leads to the diffusion of amino acids inside the melted spots and their coupling to the surface.

Exploiting combinatorial laser fusing, peptide arrays with a spot density of 40,000 peptide spots per cm^2^ were synthesized (Figure 8d). The particles had a mean size of 2–5 µm and were composed of amino acid derivatives imbedded in an inert styrene-acrylic copolymer matrix material. In comparison to xerographic methods, no charging of particles was necessary. This fact simplified the requirements of particle composition. In combinatorial laser fusing, the size of the melted spots is directly proportional to the thickness of the particle layer (Figure 8a).

By extending the method of combinatorial laser fusing, not only peptides, but also other molecules can be synthesized in large-scale, high-density array format. The only requirement to synthesize other small molecule arrays is the development of microparticles containing the desired monomers. For instance, microparticles with embedded biotin derivatives were used to pattern biotin spots in high‑density array format. Figure 8e shows the fluorescent staining of a biotin pattern with streptavidin.

**Figure 9:**
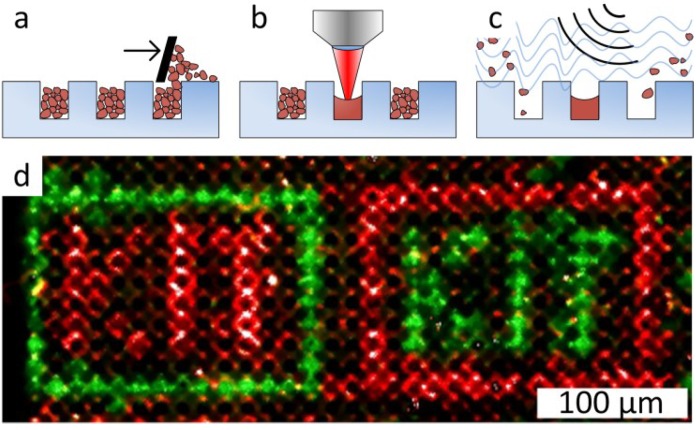
Combinatorial Laser fusing in microcavities. (**a**) Filling cavities with amino acid particles; (**b**) particle melting with laser radiation; (**c**) removal of non-melted particles with ultrasonic cleaning; (**d**) synthesis in microcavities (500,000 cavities per cm^2^, 10-µm cavity depth) with biotin and cysteine particles. Afterwards, the HA peptide was coupled from solution to the cysteine. Subsequently, biotin was labeled with streptavidin (green) and the complementary pattern with HA peptides with anti-HA-antibodies (red). Creative Commons Attribution-Share Alike 3.0 DE License (CC-BY-SA 3.0 DE) [55].

In follow-up experiments, glass substrates with microcavities for selective laser fusing help to avoid the dependence of the spot size on particle layer parameters (Figure 9a). In this case, the microcavities are filled with particles just by swiping particles over the structured glass surface. After selective melting with laser radiation, non-melted particles are removed from the microcavities by ultrasonic cleaning (Figure 9b,c). Thus, the spot density is defined by the density of the microcavities on the glass substrate. A proof-of-principle synthesis in microcavities with a density of 500,000 spots per cm^2^ using combinatorial laser fusing is demonstrated in Figure 9d.

## 4. Conclusions

Particle-based microarrays have attracted a lot of attention from scientists over the last two decades, due to their enormous potential in high-throughput screening applications. Originally, particle patterning in array format was used for the probing or sequencing of oligonucleotides. In both cases, the particles act as carriers for already synthesized libraries of oligonucleotides, where each bead represents a unit of only one sequence of nucleotides. These applications became possible only because of the development of the ePCR technique and the possibility of decoding nucleotide sequences after the oligonucleotides were already linked to beads.

As efficient possibilities are still missing in the case of oligopeptides, particle-based peptide arrays are synthesized according to a different principle: particles are used as carriers of amino acid derivatives, which are imbedded inside a polymer matrix. After particle delivery to the synthesis areas, they are melted, the monomers are coupled and the particle matrix is afterwards removed from the array surface. Combinatorial laser fusing allows for the synthesis of particle-based peptide arrays with densities comparable to oligonucleotide arrays. Combined with microwells, this technique leads to further improvements in array density, which is only dependent on the density of the microcavities. In contrast to particles in biofunctional xerography, which have to contain charge activating and charge stabilizing agents, melting of particles occurs without particle charging, simplifying particle composition and production. Finally, in a proof-of-principle synthesis with particles in microwells, an unprecedented density of 500,000 spots/cm^2^ was demonstrated.

As the example synthesis of the biotin pattern shows, combinatorial laser fusing can also be extended to the synthesis of other molecules, not only peptides. In material and life sciences, this opens up the road to high-density arrays with any artificial molecules, which considerably increases the screening capacity of combinatorial libraries. Especially in life sciences, cost-efficient high-density peptide arrays represent an attractive method for high-throughput identification of peptide-protein interactions.
